# Quantum-enabled temporal and spectral mode conversion of microwave signals

**DOI:** 10.1038/ncomms10021

**Published:** 2015-11-30

**Authors:** R. W. Andrews, A. P. Reed, K. Cicak, J. D. Teufel, K. W. Lehnert

**Affiliations:** 1 JILA, University of Colorado and NIST, Boulder, Colorado 80309, USA; 2 Department of Physics, University of Colorado, Boulder, Colorado 80309, USA; 3 National Institute of Standards and Technology (NIST), Boulder, Colorado 80305, USA

## Abstract

Electromagnetic waves are ideal candidates for transmitting information in a quantum network as they can be routed rapidly and efficiently between locations using optical fibres or microwave cables. Yet linking quantum-enabled devices with cables has proved difficult because most cavity or circuit quantum electrodynamics systems used in quantum information processing can only absorb and emit signals with a specific frequency and temporal envelope. Here we show that the temporal and spectral content of microwave-frequency electromagnetic signals can be arbitrarily manipulated with a flexible aluminium drumhead embedded in a microwave circuit. The aluminium drumhead simultaneously forms a mechanical oscillator and a tunable capacitor. This device offers a way to build quantum microwave networks using separate and otherwise mismatched components. Furthermore, it will enable the preparation of non-classical states of motion by capturing non-classical microwave signals prepared by the most coherent circuit quantum electrodynamics systems.

## Abstract (web summary)

Cavity QED systems which can be used for quantum information processing can absorb or emit signals with specific frequencies and temporal envelops. Here, the authors show that the temporal and spectral content of microwave signals can be manipulated with a flexible aluminium drumhead embedded in a circuit.

## Graphical abstract

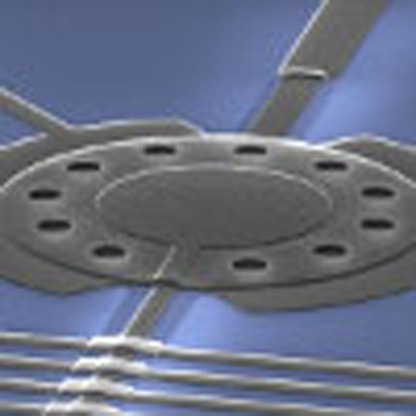

Cavity quantum electrodynamics (QED) and its low-frequency on-chip counterpart circuit QED form prototypical quantum systems that have enabled many tests of quantum theory and advanced goals of quantum computation^1^,^2^. A single cavity QED (cQED) system links a quantum two-level system (qubit) to excitations of the electromagnetic field. Linking multiple, spatially separated cQED systems with electromagnetic waves provides a scalable quantum networking architecture that could combine a large number of individual systems (or nodes) into one network^3^,^4^,^5^. Deterministically distributing information between different nodes requires the electromagnetic signals generated by one node be completely absorbed by another. This requirement can be viewed as a generalized impedance matching condition, which can be satisfied by controlling the temporal and spectral content of the signal^6^,^7^.

The content of an electromagnetic signal can be controlled using a suitable nonlinearity. For example, at microwave frequencies, the nonlinear inductance provided by Josephson junctions has been used to shape the temporal envelope of signals^8^,^9^,^10^,^11^ or alter their frequency^12^,^13^,^14^. Apart from Josephson junctions, the nonlinear interaction between electricity (or light) and a vibrating mass has been used to store electromagnetic signals^15^,^16^,^17^ and convert them between different frequencies^18^,^19^,^20^. However, no scheme has yet demonstrated arbitrary temporal and spectral control of electromagnetic signals using a single device.

In this work, we combine microwave-frequency pulse shaping and conversion between adjustable frequencies by embedding a mechanical oscillator in a tunable circuit. As a demonstration of our temporal and spectral mode converter, we use it to implement a particular protocol that converts the temporal envelope of a 7 GHz microwave signal from a decaying exponential to a Gaussian, and also shifts the signal's frequency by up to 250 MHz. Such a protocol can process signals emitted from coherent circuit QED systems, which emit signals in a fixed, narrow (≲10 MHz) frequency range, and with an exponentially decaying envelope^21^,^22^. To test the suitability of the mode converter for use with quantum signals, we characterize the noise acquired by low-amplitude classical signals during temporal and spectral mode conversion. We find the total added noise is less than one quantum for frequency shifts up to approximately 100 MHz, and ∼0.4 quanta for small frequency shifts, indicating that the mode converter approaches the performance required to preserve fragile quantum signals. (If the added noise is larger than half a quantum, negative features of a signal's Wigner quasi-probability distribution vanish^23^.)

## Results

### Tunable electromechanical circuit

**Figure 1:**
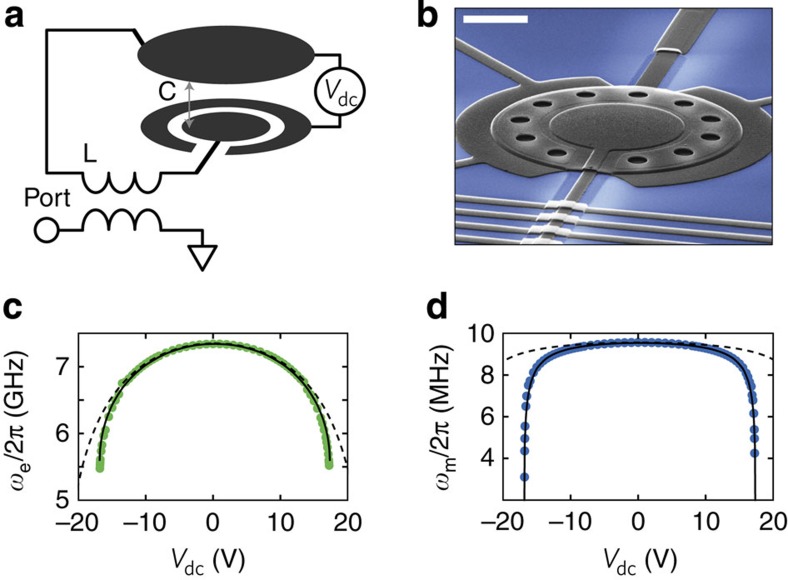
A tunable electromechanical circuit. (**a**) An inductor (L) and a parallel plate capacitor (C) form a microwave-frequency electromagnetic resonator that is inductively coupled to a transmission line (Port). The upper plate of the parallel plate capacitor is clamped only on the edges, and is free to vibrate like a drumhead. The capacitance of the microwave circuit can be tuned by applying a voltage (*V*_dc_) between an annular electrode and the drumhead. (**b**) A scanning electron micrograph of the parallel plate capacitor, formed by sputtering layers of aluminium (dark grey) onto a sapphire substrate (blue); the micrograph is oriented similarly to **a**. Scale bar is 5 μm in length. (**c**,**d**) Measured microwave resonant frequency (*ω*_e_) and mechanical resonant frequency (*ω*_m_) as a function of *V*_dc_. Solid lines show the expected performance as calculated with a finite element simulation that includes an attractive force that scales like the Casimir force and is within a factor of two of an estimated Casimir force magnitude^29^; neglecting this additional attractive force results in much different behaviour shown with the dashed lines (Supplementary Note 1, Supplementary Figs 1 and 2). Although incorporation of the Casimir force accounts for our data, we cannot rule out some contribution from patch potentials^30^.

The mode converter relies on a mechanical oscillator formed by a flexible aluminium drumhead, as shown in Fig. 1 (ref. 24). The drumhead is an integral part of a microwave circuit that is addressed with a coaxial transmission line. During operation, the circuit is mounted on the base of a dilution refrigerator, and cooled to <25 millikelvin. Applying a voltage, *V*_dc_, between the drumhead and an additional annular electrode (Fig. 1a) creates an attractive force between the two metal films. As the drumhead is free to move, it deflects and increases the total capacitance of the microwave circuit, and thus lowers its resonant frequency, *ω*_e_, as shown in Fig. 1c. The separation between the drumhead and microwave electrode is ∼40 nm with *V*_dc_=0, and at this separation each nanometre of drumhead motion alters the resonant frequency of the microwave circuit by 42 MHz, or an electromechanical coupling of *G*=2*π* × 42 MHz nm^−1^. By using the electrostatic force to move the drumhead ∼20 nm, we shift the circuit's resonant frequency by over 1 GHz, much more than the circuit's bandwidth of *κ*=2*π* × 2.5 MHz. As *V*_dc_ is increased, the electrostatic force and other attractive forces eventually overwhelm the restoring force provided by tension in the drumhead, evidenced by the decrease in the vibrational frequency, *ω*_m_, as shown in Fig. 1d. When the vibrational frequency reaches zero, the attractive forces dominate and the drumhead will collapse.

Vibrations of the drumhead are also affected by the electromechanical coupling *G*. At *V*_dc_=0, *G*=2*π* × 42 MHz nm^−1^, and in the presence of a detuned microwave pump, this coupling exchanges excitations of the microwave circuit and vibrational excitations of the drumhead at a rate 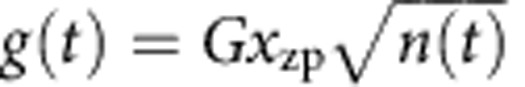 (ref. 16), where *x*_zp_=6.4 fm is the zero-point motion of the mechanical oscillator and *n*(*t*) is the strength of the microwave pump expressed as the number of photons induced in the circuit by the pump. We choose to work in the weak-coupling regime, where excitations in the microwave circuit decay into the transmission line (see Fig. 1a) at a rate *κ*_ext_>2*g*. In this regime, the microwave circuit simply enhances an exchange between excitations in the transmission line and vibrational excitations of the drumhead^25^; this exchange occurs at a rate Γ(*t*)=4*g*(*t*)^2^/*κ*, where *κ* is the total energy decay rate of the microwave circuit. The controllable, time-dependent coupling to a transmission line provided by this device is a basic requirement for controlling the temporal envelope of signals^7^.

### Mode conversion protocol

**Figure 2:**
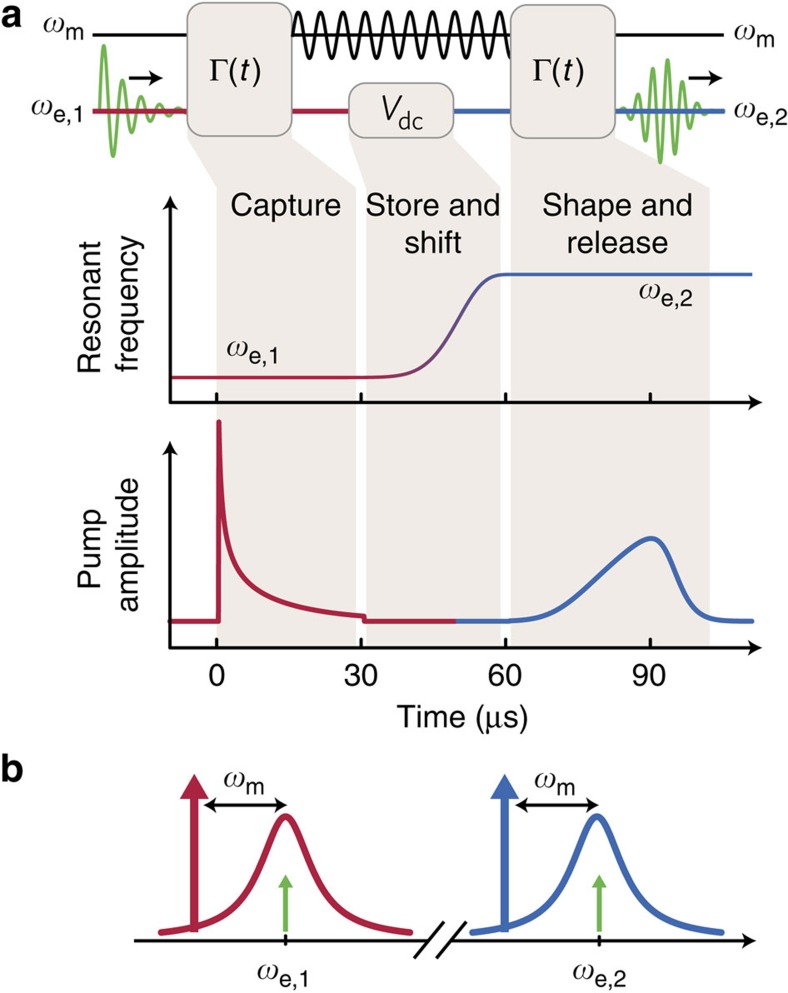
Protocol for mode conversion. (**a**) A signal in the transmission line at frequency *ω*_e,1_ (green sinusoid) is first converted into a vibration of the aluminium drumhead (black sinusoid). Γ(*t*) is adjusted by varying the power in a pump at frequency *ω*_e,1_−*ω*_m_ (red waveform). Then we alter the frequency of the microwave circuit by changing *V*_dc_. During this time, the signal is stored in the vibrating drumhead; the change in *V*_dc_ adiabatically changes *ω*_m_ by approximately 2*π* × 300 kHz. Finally, the signal is transferred from the drumhead back into the transmission line at frequency *ω*_e,2_ by adjusting the power in a pump at frequency *ω*_e,2_−*ω*_m_ (blue waveform). (**b**) Frequency domain schematic of mode conversion. Microwave pumps (red and blue arrows) applied at a frequency *ω*_m_ below the microwave circuit (response shown as the red and blue curves) allow a signal at frequency *ω*_e,1_ to be converted to a signal at frequency *ω*_e,2_ (green arrows).

Our protocol for mode conversion consists of modulating both *V*_dc_ and Γ(*t*). Initially, we modulate Γ(*t*) to capture a microwave signal propagating down the transmission line and store it as a vibration of the drumhead^26^. The signal can be stored as a vibration for over (*n*_th_*κ*_m_)^−1^≈180 μs before it gains one quantum of energy from the environment. This storage time is sufficient to complete the mode conversion protocol, and can be adjusted to serve as a signal delay. Once the signal is captured, we change *V*_dc_ to alter the microwave circuit's resonant frequency. We then modulate Γ(*t*) to release the signal stored in the drumhead back into the transmission line. Even though the released signal has different spectral and temporal content from the initial signal, the information in the initial signal has ideally been preserved. The protocol is schematically depicted in Fig. 2.

Signals reflected or emitted from the mode converter are measured with a microwave receiver that consists of a Josephson parametric amplifier^27^ followed by additional amplifiers, a down-converting mixer and a digitizer (Supplementary Note 2, Supplementary Fig. 3). Collected data consist of time-stamped voltage values that we coherently average by running multiple repetitions of the mode conversion protocol. To clearly represent the time and frequency content of a measured voltage waveform, we construct the Wigner–Ville distribution using the discretized version of 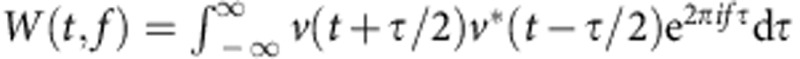, where *W*(*t*, *f*) is the Wigner–Ville distribution and *v*(*t*) is the analytic voltage measured at time *t*. The analytic voltage is constructed using a Hilbert transform on the measured voltage^28^: 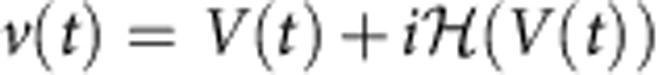, where *V*(*t*) is the voltage measured at time *t* and 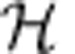 is the Hilbert transform. The resulting time-frequency distribution is related to the position-momentum Wigner quasi-probability distribution; however, nothing ‘quantum' is associated with a negative distribution. The marginal distributions of the Wigner–Ville distribution give the temporal envelope and energy spectral density of the voltage waveform.

### Mode conversion results

**Figure 3:**
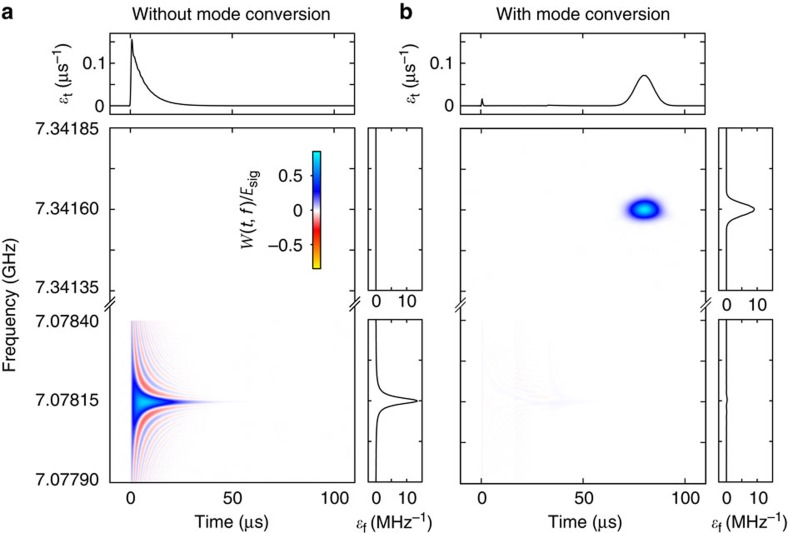
Temporal and spectral mode conversion of a microwave signal. (**a**) Wigner–Ville distribution of a signal that reflects off the mode converter (so no mode conversion is performed) and is measured with a microwave receiver. The marginal distributions of the Wigner–Ville distribution show the signal is a decaying exponential in time, and a Lorentzian in frequency; 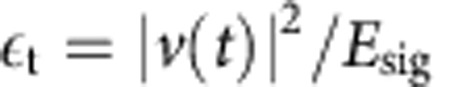 and 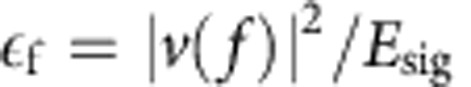. The signal is centred near 7.07815 GHz and has a power decay rate of 2*π* × 24 kHz. There is no signal content near 7.34160 GHz. (**b**) Wigner–Ville distribution of a mode-converted signal. When the mode converter is used, the decaying exponential signal is captured as a vibration of the drumhead, and the signal content at 7.07815 GHz is almost entirely absent. Later in time, we recover the signal that is now centred near 7.34160 GHz and has a Gaussian temporal envelope and Gaussian spectral content with width 2*π* × 24 kHz.

As an initial test of our measurement apparatus, we generate a signal near 7.08 GHz that has an exponentially decaying envelope (power decay rate of 2*π* × 24 kHz) and inject it into the coaxial transmission line that connects to the mode converter. We choose a signal with this temporal envelope because it resembles the temporal envelope of quantum signals emitted from highly coherent circuit QED systems^21^. We initially set *V*_dc_ so that the microwave circuit has a resonant frequency substantially different from the injected signal, and the signal reflects off the mode converter and is measured by our microwave receiver. The measured Wigner–Ville distribution of the injected signal is shown in Fig. 3a, with the data normalized by 
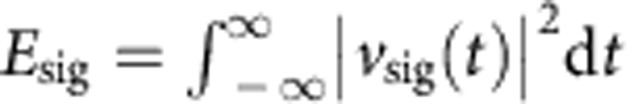
, the total energy in the unconverted signal.

To perform mode conversion, we set *V*_dc_≈10 V so the microwave circuit is centred near 7.08 GHz, and send in the signal with the decaying exponential envelope. We apply a microwave pump with a time-dependent amplitude as shown in Fig. 2a to alter Γ(*t*). The pump amplitude is carefully chosen^26^ so that we capture almost all of the signal and store it as a vibration of the drumhead (Supplementary Note 3, Supplementary Fig. 4). Only a small amount (∼4%) of the signal energy is reflected out of the converter, as demonstrated in Fig. 3b. We then set *V*_dc_=0 so that the microwave circuit is centred near 7.34 GHz, and again apply a microwave pump with a time-dependent amplitude as shown in Fig. 2a. The Wigner–Ville distribution of the signal emitted by the mode converter is plotted in Fig. 3b. From the relative energies in the reflected and converted signals, we find the photon number efficiency of the conversion process to be 0.81±0.03, in good agreement with an expected efficiency of (*κ*_ext_/*κ*)^2^ × 0.96=0.81±0.04 where 0.96 is the efficiency with which we capture the injected signal^26^.

The mode converter can be used to change signals between arbitrarily chosen temporal and spectral modes. We demonstrated frequency conversion over approximately 250 MHz; conversion over a larger window is feasible but the mode converter becomes more sensitive to fluctuations in *V*_dc_ at higher bias. Although we altered the temporal envelope of a microwave signal from a decaying exponential to a Gaussian, any other envelope is possible, limited only by the bandwidth of the mode converter. The maximum bandwidth is set by the rate at which information can be exchanged between the transmission line and the drumhead, which for this device is limited by the microwave circuit's bandwidth of *κ*/2≈2*π* × 1.3 MHz. The minimum bandwidth is set by the mechanical decoherence rate. Even though energy in the drumhead is lost to the environment at the modest rate of *κ*_m_=2*π* × 25 Hz, fluctuations of the environment incoherently drive vibrations of the drumhead. To avoid these environment-driven vibrations, signals must be processed faster than the mechanical decoherence rate of *n*_m_*κ*_m_=2*π* × 900 Hz, where *n*_m_ is the thermal equilibrium phonon occupation number of the drumhead (Supplementary Note 4, Supplementary Fig. 5, Supplementary Table 1). Avoiding these excess vibrations is essential when manipulating fragile quantum signals.

**Figure 4:**
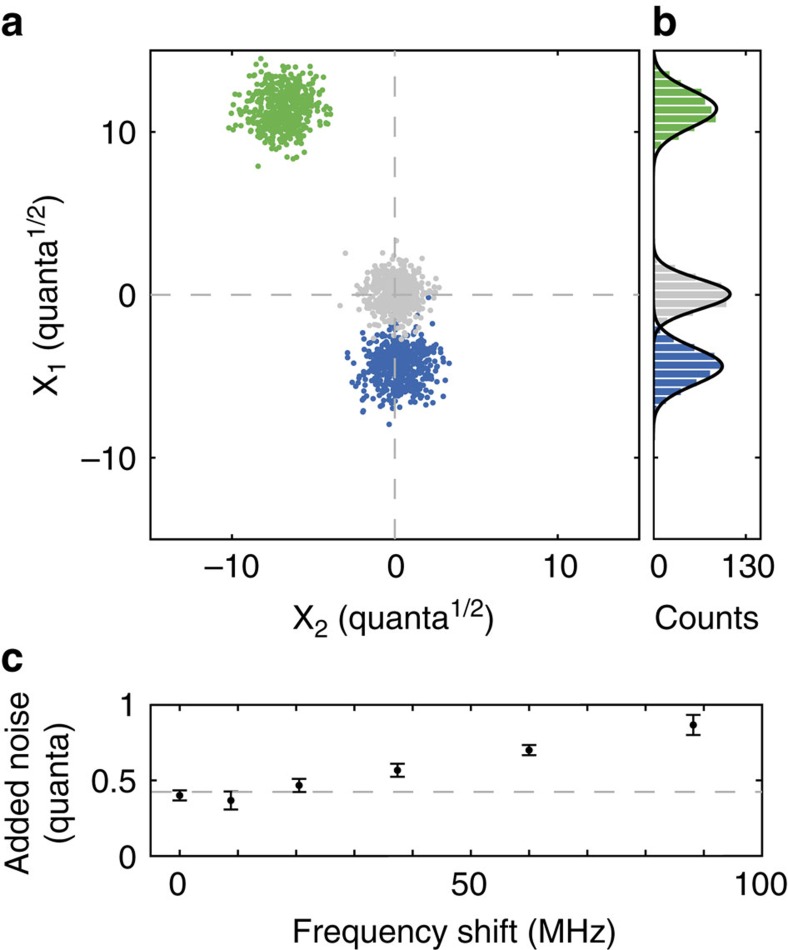
Noise added during mode conversion. (**a**) Quadrature amplitudes for 500 independent repetitions of the mode conversion protocol, found using least-squares parameter estimation (Supplementary Note 5). Results of conversion with a 90 MHz frequency shift with a signal containing ∼10 quanta (blue points) and ∼100 quanta (green points) of energy, which demonstrates the dynamic range of the converter. Low- and high-amplitude signals are intentionally generated with different phases. For reference, a vacuum input into our microwave receiver yields the grey points. The total variance of the quadrature amplitudes, Var(*X*_1_)+Var(*X*_2_), subtracted from the variance of measured vacuum indicates the amount of noise added during signal preparation and mode conversion, which we find to be 0.9±0.1 quanta. (**b**) *X*_1_ marginal of the quadrature amplitudes with Gaussian fits (black curves). (**c**) For frequency shifts less than 90 MHz, we observe less added noise. Error bars, s.e.m. Dashed grey line indicates expected added noise from a mechanical decoherence rate of *n*_m_*κ*_m_=2*π* × 900 Hz.

To verify that our converter operates quickly and quietly enough to avoid corrupting signals, we collect statistics on single-shot measurements of mode conversion using low-amplitude (∼10 quanta of energy) signals. We inject a low-amplitude signal with a decaying exponential envelope into the converter, shift its frequency by 90 MHz, and recover a signal with a Gaussian envelope. The central frequency and temporal envelope of the converted signal are already known, and we use this information to perform a least-squares parameter estimation of the quadrature amplitudes *X*_1_ and *X*_2_ (Supplementary Notes 5 and 6, Supplementary Fig. 6, Supplementary Tables 2 and 3). The quadrature amplitudes are two independent values that can be recast as the amplitude and phase of the converted signal. The result of each single-shot measurement and quadrature amplitude estimation appears as a single point in Fig. 4a. The scatter of the quadrature amplitudes indicates the total amount of noise added to the signal during preparation, mode conversion, and measurement. By comparing the total variance, Var(*X*_1_)+Var(*X*_2_), of the converted signal with that of measured vacuum noise, we infer the mode converter adds 0.9±0.1 quanta during conversion with a 90 MHz frequency shift, where the uncertainty is the s.e.m. of the inferred added noise for three consecutive data sets, each set formed by running 500 repetitions of the protocol. For mode conversion with frequency shifts ≲20 MHz, we observe an added noise of ≲0.5 quanta (Fig. 4c), a value that we entirely attribute to environment-driven vibrations of the drumhead.

## Discussion

We have demonstrated a temporal and spectral mode converter whose operating frequency, bandwidth, and low noise properties make it compatible with many circuit QED systems. As a next step, our mode converter can be integrated with a single cQED system consisting of a superconducting qubit embedded in a high-quality microwave resonator. This simple network could serve as a prototype for larger microwave quantum networks. Beyond quantum networking, our mode converter can capture non-classical microwave signals, enabling the preparation of non-classical motional states in a macroscopic object.

## Additional information

**How to cite this article:** Andrews, R. W. *et al*. Quantum-enabled temporal and spectral mode conversion of microwave signals. *Nat. Commun.* 6:10021 doi: 10.1038/ncomms10021 (2015).

## Supplementary Material

Supplementary Information Supplementary Figures 1-6, Supplementary Tables 1-3, Supplementary Notes 1-6 and Supplementary References

## References

[b1] Miller R. . Trapped atoms in cavity QED: coupling quantized light and matter. J. Phys. B: At. Mol. Opt. Phys. 38, S551 (2005).

[b2] Schoelkopf R. J. & Girvin S. M. Wiring up quantum systems. Nature 451, 664–669 (2008). 18256662 10.1038/451664a

[b3] O'Brien J. L., Furusawa A. & Vuckovic J. Photonic quantum technologies. Nat. Photon. 3, 687–695 (2009).

[b4] Kimble H. J. The quantum internet. Nature 453, 1023–1030 (2008). 18563153 10.1038/nature07127

[b5] Ritter S. . An elementary quantum network of single atoms in optical cavities. Nature 484, 195–200 (2012). 22498625 10.1038/nature11023

[b6] Cirac J. I., Zoller P., Kimble H. J. & Mabuchi H. Quantum state transfer and entanglement distribution among distant nodes in a quantum network. Phys. Rev. Lett. 78, 3221–3224 (1997).

[b7] Korotkov A. N. Flying microwave qubits with nearly perfect transfer efficiency. Phys. Rev. B 84, 014510 (2011).

[b8] Pechal M. . Microwave-controlled generation of shaped single photons in circuit quantum electrodynamics. Phys. Rev. X 4, 041010 (2014).

[b9] Srinivasan S. J. . Time-reversal symmetrization of spontaneous emission for quantum state transfer. Phys. Rev. A 89, 033857 (2014).

[b10] Yin Y. . Catch and release of microwave photon states. Phys. Rev. Lett. 110, 107001 (2013). 23521281 10.1103/PhysRevLett.110.107001

[b11] Pierre M., Svensson I.-M., Sathyamoorthy S. R., Johansson G. & Delsing P. Storage and on-demand release of microwaves using superconducting resonators with tunable coupling. Appl. Phys. Lett. 104, 232604 (2014).

[b12] Roch N. . Widely tunable, nondegenerate three-wave mixing microwave device operating near the quantum limit. Phys. Rev. Lett. 108, 147701 (2012). 22540823 10.1103/PhysRevLett.108.147701

[b13] Abdo B. . Full coherent frequency conversion between two propagating microwave modes. Phys. Rev. Lett. 110, 173902 (2013). 23679729 10.1103/PhysRevLett.110.173902

[b14] Inomata K. . Microwave down-conversion with an impedance-matched A system in driven circuit QED. Phys. Rev. Lett. 113, 063604 (2014). 25148329 10.1103/PhysRevLett.113.063604

[b15] Verhagen E., Deleglise S., Weis S., Schliesser A. & Kippenberg T. J. Quantum-coherent coupling of a mechanical oscillator to an optical cavity mode. Nature 482, 63–67 (2012). 22297970 10.1038/nature10787

[b16] Palomaki T. A., Harlow J. W., Teufel J. D., Simmonds R. W. & Lehnert K. W. Coherent state transfer between itinerant microwave fields and a mechanical oscillator. Nature 495, 210–214 (2013). 23486060 10.1038/nature11915

[b17] Fiore V. . Storing optical information as a mechanical excitation in a silica optomechanical resonator. Phys. Rev. Lett. 107, 133601 (2011). 22026851 10.1103/PhysRevLett.107.133601

[b18] Hill J. T., Safavi-Naeini A. H., Chan J. & Painter O. Coherent optical wavelength conversion via cavity optomechanics. Nat. Commun. 3, 1196 (2012). 23149741 10.1038/ncomms2201

[b19] Andrews R. W. . Bidirectional and efficient conversion between microwave and optical light. Nat. Phys. 10, 321–324 (2014).

[b20] Dong C., Fiore V., Kuzyk M. C. & Wang H. Optomechanical dark mode. Science 338, 1609–1613 (2012). 23160956 10.1126/science.1228370

[b21] Houck A. A. . Generating single microwave photons in a circuit. Nature 449, 328–331 (2007). 17882217 10.1038/nature06126

[b22] Santori C., Fattal D., Vuckovic J., Solomon G. S. & Yamamoto Y. Indistinguishable photons from a single-photon device. Nature 419, 594–597 (2002). 12374958 10.1038/nature01086

[b23] Leonhardt U. Measuring the Quantum State of Light Cambridge University Press (1997).

[b24] Cicak K. . Low-loss superconducting resonant circuits using vacuum-gap-based microwave components. Appl. Phys. Lett. 96, 093502 (2010).

[b25] Zhang J., Peng K. & Braunstein S. L. Quantum-state transfer from light to macroscopic oscillators. Phys. Rev. A 68, 013808 (2003).

[b26] Harlow J. W. *Microwave Electromechanics: Measuring and Manipulating the Quantum State of a Macroscopic Mechanical Oscillator*. PhD thesis, University of Colorado at Boulder (2013).

[b27] Castellanos-Beltran M. A., Irwin K. D., Hilton G. C., Vale L. R. & Lehnert K. W. Amplification and squeezing of quantum noise with a tunable Josephson metamaterial. Nat. Phys. 4, 929–931 (2008).

[b28] Boashash B. Note on the use of the Wigner distribution for time-frequency signal analysis. IEEE Trans. Acoust. Speech Signal Process 36, 1518–1521 (1988).

[b29] Intravaia F. & Lambrecht A. Surface plasmon modes and the casimir energy. Phys. Rev. Lett. 94, 110404 (2005). 15903834 10.1103/PhysRevLett.94.110404

[b30] Speake C. C. & Trenkel C. Forces between conducting surfaces due to spatial variations of surface potential. Phys. Rev. Lett. 90, 160403 (2003). 12731962 10.1103/PhysRevLett.90.160403

