# Supplementary material for: Quantum-enabled temporal and spectral mode conversion of microwave signals
Source: Nat Commun. 2015 Nov 30;6:10021. doi: 10.1038/ncomms10021 (PMC4674675; doi:10.1038/ncomms10021)
Supplement: Supplementary Information — Supplementary Figures 1-6, Supplementary Tables 1-3, Supplementary Notes 1-6 and Supplementary References [file ncomms10021-s1.pdf]

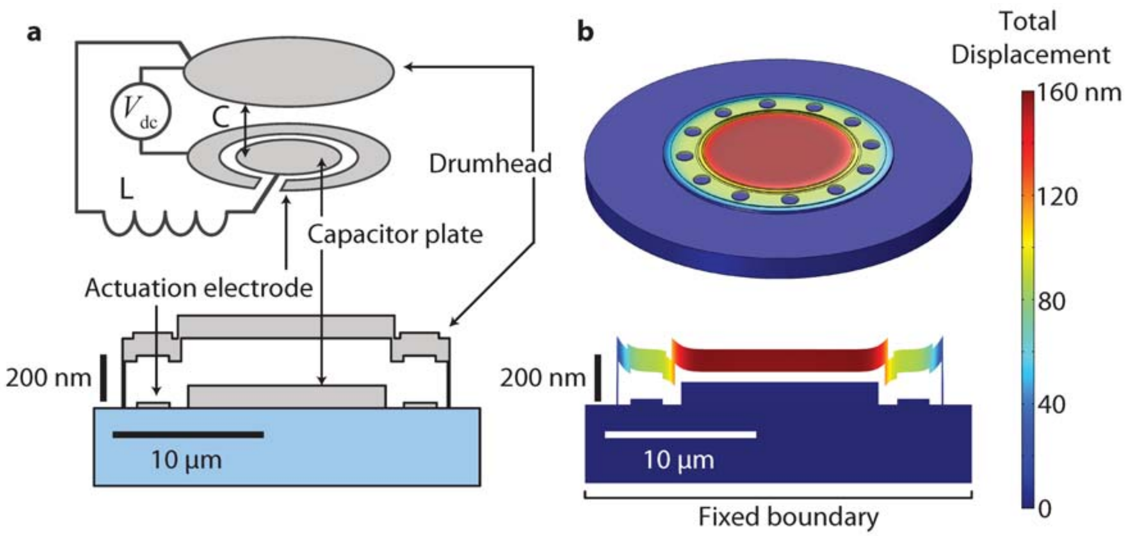

Supplementary Figure 1: Mode converter geometry. **a**, Diagram of the mode converter, and cross section of the parallel plate capacitor, which is formed by depositing aluminum films (gray) on a sapphire substrate (light blue). At room temperature, there is approximately 200 nm of separation between the two metal films. **b**, Finite element simulation of device geometry after cooling from room temperature to <25 mK.

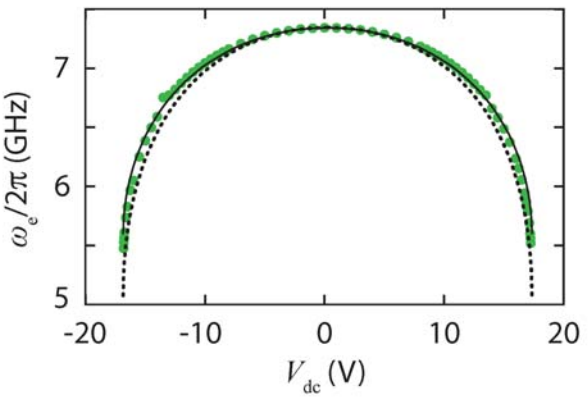

Supplementary Figure 2: Measured microwave resonant frequency (ω_e) as a function of V_{dc}. The solid line shows the predicted frequency tuning as calculated with a finite element simulation that includes a Casimir force. The dotted line shows the predicted frequency tuning as calculated with a simulation that includes a 0.75 V potential difference between the drumhead and the capacitor plate (but no Casimir force).

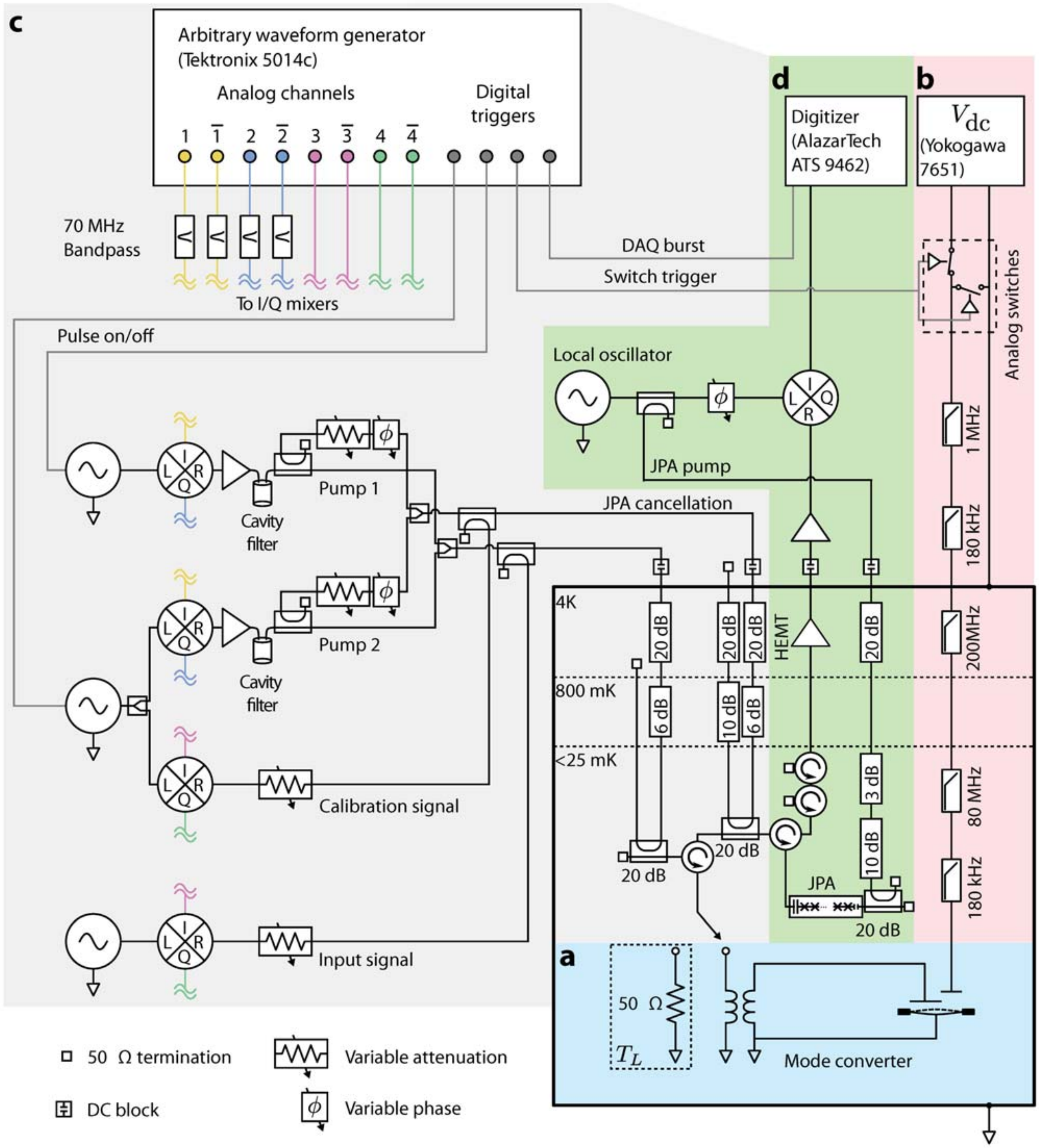

Supplementary Figure 3: Measurement network. a, Mode converter at <25 mK and load resistor at $T_{\rm L}$. **b,** Actuation line. **c,** Arbitrary waveform generation for microwave pumps and signals. **d,** Microwave receiver.

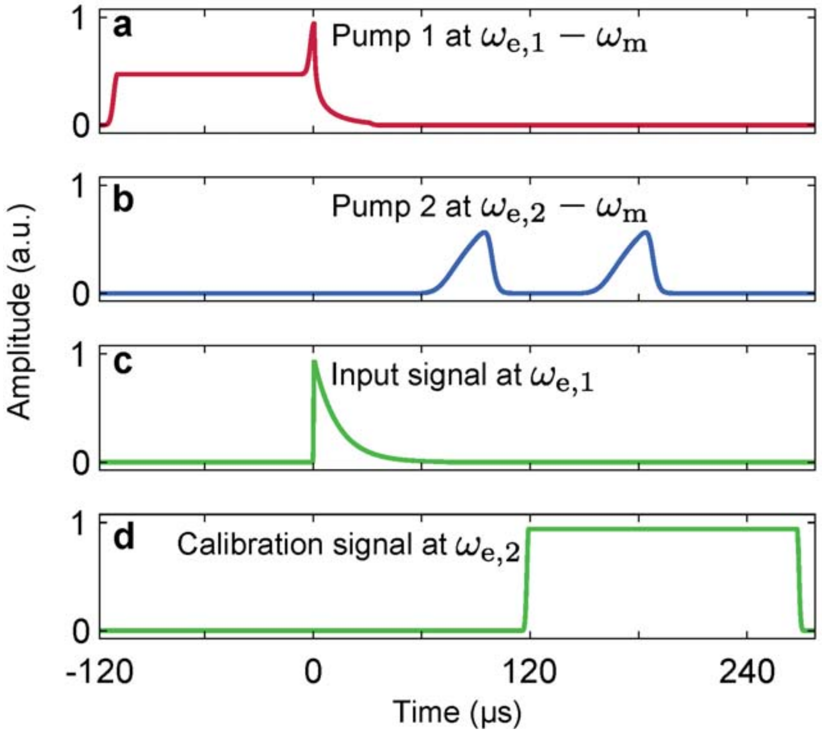

Supplementary Figure 4: Temporal envelopes of microwave pumps and signals used during temporal and spectral mode conversion. **a,** Initially, a constant pump cools the motion of the drumhead. From $0 < t < 30$ μs, the amplitude of the pump is modulated so that the coupling is given by Eqn. 4. **b,** After a 30 μs storage time ($30 < t < 60$ μs), the amplitude of the pump is modulated so the coupling is given by Eqn. 5. A second, identical pulse is used in conjunction with the calibration signal to ensure the pump does not saturate the JPA's gain. **c,** An exponentially decaying signal at frequency $\omega_{e,1}$ is injected into the mode converter. **d,** A calibration signal at frequency $\omega_{e,2}$ is used to monitor the JPA gain and correct for timing errors.

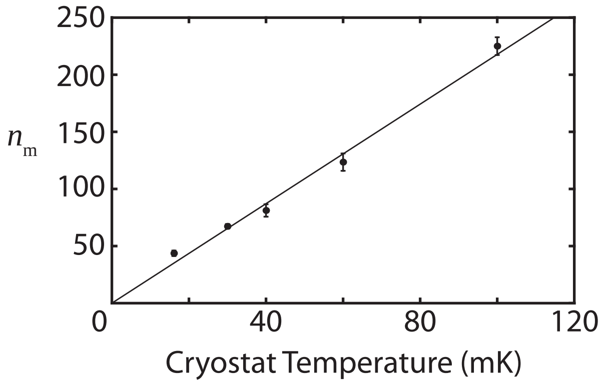

Supplementary Figure 5: Electromechanical coupling and thermalization of the aluminum drumhead. Black line shows expected phonon occupancy, n_m, given equipartition and an electromechanical coupling rate of $g_0 = 2\pi \times 270$ Hz.

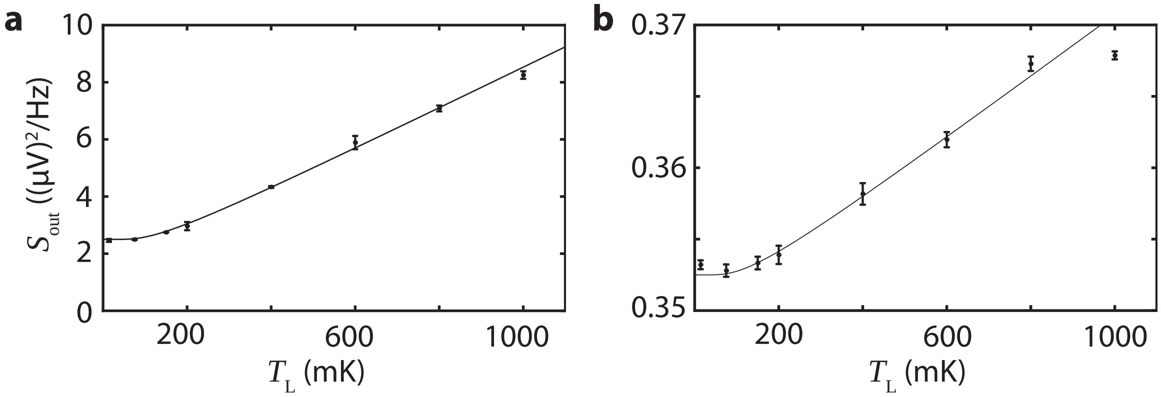

Supplementary Figure 6: Calibration of microwave receiver. The output spectral density of the microwave receiver varies with the temperature of the load resistor. **a**, A fit to Eqn. 9 (solid line) shows the receiver has an efficiency of $\zeta_1 = 0.49$ and $\mathcal{G}_1 = 5.0$ $(\mu\mathrm{V})^2/(\mathrm{Hz}\cdot\mathrm{quanta})$. **b**, Without the JPA, the efficiency of the receiver drops to $\zeta_1' = 0.0105$ and the gain reduces to $\mathcal{G}_1' = 0.705$ $(\mu\mathrm{V})^2/(\mathrm{Hz}\cdot\mathrm{quanta})$.

Supplementary Table 1: Parameters of electromechanical circuit.

Symbol	Description	Value and units
$\omega_{\mathrm{e}}/2\pi$	Circuit resonant frequency (at $V_{\mathrm{dc}}=0$)	7.34 GHz
$\kappa/2\pi$	Circuit decay rate	2.5 ± 0.1 MHz
$\kappa_{\mathrm{ext}}/2\pi$	Circuit decay rate into transmission line	$0.92{\times}\kappa/2\pi$
$\omega_{\mathrm{m}}/2\pi$	Mechanical resonant frequency (at $V_{\mathrm{dc}}=0$)	9.56 MHz
$\kappa_{\mathrm{m}}/2\pi$	Mechanical decay rate	25 ± 2 Hz
n_{m}	Average occupancy of the mechanical oscillator	36 ± 2
$n_{\mathrm{m}}\kappa_{\mathrm{m}}/2\pi$	Mechanical decoherence rate	900 ± 90 Hz
$g_0/2\pi = Gx_{\mathrm{zp}}/2\pi$	Electromechanical coupling	270 ± 5 Hz

Supplementary Table 2: Gain and efficiency of the microwave receiver.

**Operating Frequency**	**Gain** in units of $(\mu\mathrm{V})^2/(\mathrm{Hz}\cdot\mathrm{quanta})$	**Efficiency**
$\omega_{\mathrm{e},1} = 2\pi\times$ 7.07825 GHz	$\mathcal{G}_1 = 5.0 \pm 0.1$	$\zeta_1 = 0.49 \pm 0.02$
$\omega_{\mathrm{e},2} = 2\pi\times$ 7.34135 GHz	$\mathcal{G}_2 = 11.0 \pm 0.1$	$\zeta_2 = 0.60 \pm 0.02$

Supplementary Table 3: Gain and efficiency of the microwave receiver without the JPA.

**Operating Frequency**	**Gain** in units of $(\mu\mathrm{V})^2/(\mathrm{Hz}\cdot\mathrm{quanta})$	**Efficiency**
$\omega_{\mathrm{e},1} = 2\pi\times$ 7.07825 GHz	$\mathcal{G}_1' = 0.71 \pm 0.04$	$\zeta_1' = 0.011 \pm 0.001$
$\omega_{\mathrm{e},2} = 2\pi\times$ 7.34135 GHz	$\mathcal{G}_2' = 0.98 \pm 0.04$	$\zeta_2' = 0.013 \pm 0.001$

SUPPLEMENTARY NOTE 1: EXPECTED FREQUENCY TUNING OF MICROWAVE CIRCUIT

The approximate geometry of the aluminum drumhead is outlined in Supplementary Figure 1a. With this geometry, we use a finite element simulation to predict the effect of thermal contraction between the aluminum and the sapphire substrate. In simulation, the total thermal contraction is adjusted so that the central portion of the drumhead deflects by ∼160 nm, leaving a ∼40 nm separation between the plates of the microwave capacitor, a value set by the measured $g_0 = 2\pi \times 270$ Hz. The total thermal contraction needed to create this amount of deflection is seven parts per thousand, a value approximately consistent with that of bulk aluminum. The predicted effect of thermal contraction on device geometry is shown in Supplementary Figure 1b.

The predicted thermal contraction of Supplementary Figure 1b shows approximately 120 nm separates the drumhead from the actuation electrode used to deflect the drumhead. Using this thermally-altered geometry, we again use a finite element simulation to predict the effect of an applied voltage on drumhead deflection and resonant frequency. As the drumhead deflects with increasing voltage, it alters the capacitance in the microwave circuit and thus its resonant frequency. This effect is modeled by assuming a parallel plate capacitance (C_m) that is modulated by changes in the position of the drumhead, and an additional parasitic capacitance (C_p) provided by, for example, the coils of the inductor. With no applied voltage, $C_\mathrm{m}/\left(C_\mathrm{p} + C_\mathrm{m}\right) = 0.65$, estimated from electromagnetic simulations of the microwave circuit. Using this information, we predict the frequency tuning of the device shown as dashed lines in Fig. 1a and Fig. 1b of the main text.

In order to correctly predict the frequency tuning of the device, we must include the effects of the Casimir force. We include an attractive pressure of approximately $0.7 \times P_\mathrm{cas}$ in a finite element simulation of the membrane in the presence of a DC voltage, where $P_\mathrm{cas} = -\hbar c \pi^2/240 d^4$ is the Casimir pressure for two parallel, perfectly conducting plates separated by a distance d of vacuum. (For imperfectly conducting plates, a reduction in the Casimir force for small separations $d \lesssim \lambda_\mathrm{p}$ is expected, where λ_p is the plasma wavelength of the conducting plates [1].) With this additional pressure, we predict the frequency tuning of the device shown as solid lines in Fig. 1a and Fig. 1b of the main text.

Although inclusion of a Casimir force explains our data reasonably well, attractive forces

other than the Casimir force could potentially explain the observed frequency tuning of the device. One such attractive force is provided by patch potentials [2]. A finite element simulation that includes a 0.75 V potential difference between the drumhead and the capacitor plate predicts a frequency tuning shown by the dotted line in Supplementary Figure 2. This prediction does not explain our data as well as inclusion of an attractive force that scales like the Casimir force. However, the observed frequency tuning of the device could be explained by a combination of both the Casimir force and forces from patch potentials.

SUPPLEMENTARY NOTE 2: MEASUREMENT NETWORK

Supplementary Figure 3 shows a detailed diagram of the measurement network. The network consists of four main parts: The mode converter and a load resistor are mounted to the base stage of a dilution refrigerator (Supplementary Note 2A). A voltage bias on an actuation line controls the center frequency of the mode converter (Supplementary Note 2B). Microwave signals control and probe the mode converter (Supplementary Note 2C). A microwave receiver measures signals after they have interacted with the mode converter (Supplementary Note 2D).

A: Electromechanical circuit and load resistor

The mode converter and a load resistor are mounted to the base stage of a dilution refrigerator and cooled to $<$25 mK. A low insertion loss ($<$0.30 dB) 2-way switch (Radiall R577433000) connects the microwave receiver to either the mode converter or the load resistor. The temperature of the load resistor, T_{L}, can be varied without significantly affecting the temperature of the mode converter or fridge, as the load resistor has a weak thermal link to the base of the dilution refrigerator and has an attached heating element. The load resistor injects a known amount of noise into the microwave receiver, and this noise allows us to calibrate the efficiency and gain of the microwave receiver (see Supplementary Note 6).

B: Actuation line

The voltage on the actuation line controls the microwave circuit's resonant frequency. We use a stable voltage source (Yokogawa 7651) to provide a constant V_{dc}. Solid state switches (Maxim Integrated 313) change the voltage on the actuation line between V_{dc} and 0 V. The state of the switch network is controlled by a digital output of the AWG.

The actuation line is filtered to ensure voltage fluctuations in V_{dc} do not incoherently drive the mechanical oscillator. In particular, the mechanical oscillator is sensitive to voltage fluctuations near its resonant frequency of $\omega_{\mathrm{m}} \approx 2\pi \times 9$ MHz. Room temperature filters attenuate fluctuations in V_{dc} by more than 100 dB at frequencies near ω_{m}, and cryogenic low-pass filters provide additional attenuation near ω_{m}. These filters are designed so that the voltage noise seen by the mode converter (at frequencies near ω_{m}) is ideally given by Johnson noise from a few Ohms at <25 mK. Additionally, the filters provide enough bandwidth to quickly (<30 μs) change the voltage on the actuation line.

To verify voltage fluctuations in V_{dc} do not add significant noise during mode conversion, we operated the experiment with the mode converter biased to a constant V_{dc} (i.e., no frequency shift). For a constant $V_{\mathrm{dc}} \approx 10$ V, we measured an added noise of about 0.4 quanta, which is consistent with the measured added noise when $V_{\mathrm{dc}} = 0$ V.

C: Arbitrary microwave waveform generation

Waveforms with programmable amplitude, phase, and frequency are generated by a Tektronix 5014c arbitrary waveform generator (AWG). However, the AWG can only produce waveforms with center frequencies up to approximately 300 MHz. We must upconvert these waveforms so they interact with the mode converter, which has a center frequency of approximately 7 GHz. Upconversion is accomplished with single-sideband modulation, a process where the original waveform (centered at frequency ω_1) modulates a constant microwave tone (at frequency ω_2) to produce a final waveform (centered at frequency $\omega_1 + \omega_2$). Agilent PSG signal generators provide the constant microwave tones, and microwave mixers (Marki Microwave IQ4509-MXP) combine the microwave tones and the waveforms from the AWG. The relative phase, amplitude, and DC offsets of the AWG waveforms are adjusted to compensate for non-idealities in the mixer.

Channels 1 and 2 of the AWG produce four waveforms centered at 70 MHz that create the microwave pumps used during our mode conversion protocol. Bandpass filters (KR Electronics 2657) at the outputs of the AWG attenuate phase and amplitude noise by >40 dB for frequencies outside their passband (≈20 MHz centered at 70 MHz). Although the filters limit the bandwidth of waveforms produced by the AWG, this bandwidth is sufficient for our mode conversion protocol. Once the waveforms are upconverted using single-sideband modulation, they are amplified (Mini-Circuits ZVA-183V) and additionally filtered [3]. Ideally, this procedure results in a microwave pump at frequency $\omega_{\mathrm{e},1} - \omega_{\mathrm{m}}$ or frequency $\omega_{\mathrm{e},2} - \omega_{\mathrm{m}}$ that is >170 dB more powerful than at frequency $\omega_{\mathrm{e},1}$ or $\omega_{\mathrm{e},2}$. This large dynamic range is necessary to produce a powerful microwave pump whose phase and amplitude noise do not incoherently drive vibrations of the mechanical oscillator. When no pump is necessary, the Agilent PSGs are pulsed off so that they emit no microwave tone.

Channels 3 and 4 of the AWG produce waveforms centered at 30 MHz that create the signals used during our mode conversion protocol. Once upconverted, the waveforms produced by the AWG are at frequency $\omega_{\mathrm{e},1}$ or $\omega_{\mathrm{e},2}$. These signals are neither amplified nor filtered because the signals can be attenuated to reduce phase and amplitude noise. The signal at frequency $\omega_{\mathrm{e},1}$ is manipulated with the mode converter. The signal at frequency $\omega_{\mathrm{e},2}$ is used to monitor the gain of the JPA and additionally used to correct small (< 10 ps) timing errors that occur between the AWG and the Agilent PSGs during each repetition of our mode conversion protocol.

The temporal envelopes of the microwave pumps and signals produced by the AWG and upconversion are shown in Supplementary Figure 4.

D: Microwave receiver

Microwave signals reflected or emitted from the mode converter are measured via a sensitive microwave receiver. The first component of the receiver is a Josephson parametric amplifier (JPA), followed by additional amplifiers, a downconverting mixer, and a digitizer. A microwave pump detuned 1 MHz below the converter's resonant frequency of $\omega_{\mathrm{e},2} = 2\pi \times 7.34$ GHz drives the JPA. This pump also serves as the phase reference for the downconverting mixer. We adjust the phase of the pump entering the local oscillator (LO) port of the downconverting mixer so that the signal amplified by the JPA exits via the in-

phase channel of the mixer. This channel is sampled by a high speed digitizer (AlazarTech ATS 9462).

The JPA has a limited dynamic range, and the pumps used with the mode converter carry enough power to saturate the JPA's gain. We use variable attenuators and phase shifters to create cancellation tones that reduce the pump power incident on the JPA by 50 to 60 dB, which is sufficient for proper JPA operation. We monitor the gain of the JPA (for both gain drift and saturation) with a weak calibration signal (see Supplementary Figure 4d).

SUPPLEMENTARY NOTE 3: SHAPE OF $\Gamma(t)$

Controlling the temporal envelope of microwave signals using our converter requires optimally shaping $\Gamma(t)$. This section provides an overview of how we choose a suitable $\Gamma(t)$ and use our mode converter to capture and release microwave signals with arbitrary temporal envelopes.

For a cavity optomechanical system in the presence of a strong, monochromatic pump, the equations of motion for the position of the mechanical oscillator (described by $x = x_{\mathrm{zp}}(c+c^*)$) and the fluctuations of the resonator field about the pump (described by complex mode amplitude a) are [4]

$$\begin{aligned}\dot{a}(t) &= (i\Delta - \kappa/2)a(t) - ig(c(t) + c^*(t)) + \sqrt{\kappa_{\mathrm{ext}}}a_{\mathrm{in}}(t) \\ \dot{c}(t) &= (-i\omega_{\mathrm{m}} - \kappa_{\mathrm{m}}/2)c(t) - i\left(ga^*(t) + g^*a(t)\right) \\ a_{\mathrm{out}}(t) &= \sqrt{\kappa_{\mathrm{ext}}}a(t) - a_{\mathrm{in}}(t)\end{aligned} \quad (1)$$

where Δ is the difference between the pump frequency and the microwave circuit's resonant frequency, and other parameters are as given in Supplementary Table 1 and discussed in Supplementary Note 4. The amplitude $a_{\mathrm{in}}(t)$ describes the signal incident on the microwave circuit, and the coupling $g = g_0\alpha$, where $|\alpha|^2$ is the number of photons induced in the microwave circuit by the pump. Crucially, the coupling g can be modulated by changing the amplitude of the pump. If we fix the detuning to $\Delta = -\omega_{\mathrm{m}}$, move into a frame rotating at the microwave circuit's resonant frequency, and make a resolved-sideband approximation ($4\omega_{\mathrm{m}} \gg \kappa$) and a weak-coupling approximation ($g \ll \kappa$), the equations of motion reduce

to [5, 6]

$$\dot{c}(t) = -\frac{1}{2}\Gamma(t)c(t) - e^{-i\psi}\sqrt{\eta\Gamma(t)}a_{\text{in}}(t)$$
$$a_{\text{out}}(t) = e^{i\psi}\sqrt{\eta\Gamma(t)}c(t) + (2\eta - 1)a_{\text{in}}(t) \tag{2}$$

where $\Gamma(t) = 4|g(t)|^2/\kappa \gg \kappa_{\text{m}}$, $\eta = \kappa_{\text{ext}}/\kappa$, and $\psi = \text{Arg}(-ig)$.

Eqns. 2 succinctly describe how an incoming signal, $a_{\text{in}}(t)$, couples to the motion of the drumhead used in our mode converter. For $\eta \approx 1$, optimally capturing an incoming signal as a vibration of the drumhead is equivalent to minimizing the reflected signal, $a_{\text{out}}(t)$. Minimizing the energy in the reflected signal with $\eta = 1$ requires a coupling $\Gamma(t)$ of [6]

$$\Gamma(t) = \frac{e^{\kappa_{\text{m}}t}|a_{\text{in}}(t)|^2}{\frac{a_{\text{in}}(0)^2}{\Gamma(0)} + \int_0^t e^{\kappa_{\text{m}}t'}|a_{\text{in}}(t')|^2 dt'} \tag{3}$$

where $\Gamma(0)$ is the coupling at $t = 0$. This coupling can be found by using the Euler-Lagrange equation to find a stationary point of the reflected energy, $\int_0^T |a_{\text{out}}(t)|^2 dt$, with $a_{\text{out}}(t)$ as given by Eqns. 2.

In Fig. 3 of the main text, we capture an incoming signal with an exponentially decaying temporal envelope (i.e., $|a_{\text{in}}| \propto \Theta(t)e^{-\gamma t/2}$, where $\Theta(t)$ is the Heaviside step function) as a vibration of the drumhead. Optimally capturing this signal requires a $\Gamma(t)$ as prescribed by Eqn. 3:

$$\Gamma(t) = \Theta(t)\frac{\gamma e^{-\gamma t}}{1 - e^{-\gamma t} + \gamma/\Gamma(0)} \tag{4}$$

where $\Gamma(0)$ is an input; $\Gamma(0) \gg \gamma$ yields a high capture efficiency. We use $\gamma = 2\pi \times 24$ kHz and $\Gamma(0) \approx 2\pi \times 500$ kHz. Ultimately, $\Gamma(t)$ is limited by the microwave circuit's bandwidth to $\Gamma(t) < \kappa/2$.

Once the incoming signal is captured, it is temporarily stored and then released. During storage, $\Gamma(t) = 0$. To release a signal with a Gaussian temporal envelope centered at time t_{o} (i.e., $|a_{\text{out}}(t)| \propto e^{-\gamma^2(t-t_{\text{o}})^2}$), we use Eqn. 3 to determine the optimal choice of $\Gamma(t)$:

$$\Gamma(t) = \frac{\gamma e^{-\gamma^2(t-t_{\text{o}})^2}}{1 - \text{erf}\left(\gamma(t - t_{\text{o}})\right) + \delta} \tag{5}$$

where erf(t) is the error function, $\gamma = 2\pi \times 24$ kHz and $\delta \approx \gamma/\Gamma(0)e^{-\gamma^2 t_{\text{o}}^2}$ [6].

SUPPLEMENTARY NOTE 4: PARAMETERS OF ELECTROMECHANICAL CIRCUIT

Parameters of the microwave circuit, detailed in Supplementary Table 1, are determined by injecting a microwave signal and sweeping it over a large range of frequencies. Fitting the reflected microwave signal allows us to extract the resonant frequency of the microwave circuit (ω_e) and the external coupling rate (κ_ext), and total energy loss rate (κ).

Properties of the mechanical oscillator are also determined using the microwave response of the device. A microwave pump is injected into the mode converter, and motion of the aluminum drumhead modulates the pump and produces modulation sidebands on the reflected pump [7]. The frequency and spectral width of the sidebands indicate the vibrational frequency of the drumhead (ω_m) and (at low microwave power) its intrinsic damping rate (κ_m). The magnitude of the sidebands indicates the coupling strength between the microwave circuit and the aluminum drumhead. The amount of power in the sideband (P_m) at a frequency ω_m above the injected microwave pump is proportional to the amount of power in the injected microwave pump (P_c) [8]:

$$\frac{P_\mathrm{m}}{P_\mathrm{c}} = \frac{g_0^2}{2}\frac{\langle x^2\rangle}{x_\mathrm{zp}^2}\frac{\kappa_\mathrm{e}^2}{\Delta^2+(\kappa_\mathrm{e}-\kappa/2)^2}\frac{1}{(\Delta+\omega_\mathrm{m})^2+(\kappa/2)^2} \tag{6}$$

where Δ is the difference between the pump frequency and the microwave circuit's resonant frequency, x_zp is the zero-point fluctuations of the drumhead, and $\langle x^2\rangle$ describes the motion of the drumhead. For a drumhead in equilibrium with the thermal environment provided by the dilution fridge, $\langle x^2\rangle = 2x_\mathrm{zp}^2 n_\mathrm{m}$, where $n_\mathrm{m} \approx k_\mathrm{B}T/\hbar\omega_\mathrm{m}$ is the average phonon occupation of the drumhead for dilution fridge temperature T.

We vary the fridge temperature and monitor the ratio $P_\mathrm{m}/P_\mathrm{c}$, expressed as an effective phonon occupation, n_m. As shown in Supplementary Figure 5, at high temperatures (50-100 mK) the drumhead is in thermal equilibrium with the fridge as evidenced by the linear relationship between fridge temperature and n_m. This relationship allows us to extract an electromechanical coupling of $g_0 = 2\pi \times 270$ Hz. At the fridge base temperature of approximately 13 mK, the phonon occupation number saturates to $n_\mathrm{m} \approx 36$.

SUPPLEMENTARY NOTE 5: QUADRATURE AMPLITUDE EXTRACTION

We can express a continuous wave signal in units of quanta using a Fourier transform of the measured voltage waveform. For such a signal, we define quadrature amplitudes at a frequency ω:

$$
\begin{aligned}
X_1 &= \sqrt{\frac{2}{\zeta\mathcal{G}}} \cdot \sqrt{\frac{\Delta t}{N}} \cdot \sum_{k=1}^{N} y(k) \cdot \cos(\omega \cdot k \cdot \Delta t) \\
X_2 &= \sqrt{\frac{2}{\zeta\mathcal{G}}} \cdot \sqrt{\frac{\Delta t}{N}} \cdot \sum_{k=1}^{N} y(k) \cdot \sin(\omega \cdot k \cdot \Delta t)
\end{aligned} \tag{7}
$$

where $y(k)$ is the measured voltage waveform of length N, $1/\Delta t$ is the sample rate, and ζ is the measurement efficiency (see Supplementary Note 6). The factor $\sqrt{2/(\zeta\mathcal{G})}$ converts the voltage waveform into units of $\sqrt{\mathrm{Hz} \cdot \mathrm{quanta}}$ at the input of the receiver (the factor of two remains because $\mathcal{G}$ was calculated using a single-sided spectral density). The quadrature amplitudes X_1 and X_2 are proportional to the real and imaginary parts of the complex Fourier amplitude, and can be considered to represent the magnitude and phase of the signal: $X_1^2 + X_2^2$ yields an estimate of the number of quanta contained in the signal, and $\tan^{-1}(X_2/X_1)$ the phase of the signal.

Eqns. 7 are appropriate when the signal is continuous and at a known frequency. However, when a signal has a particular temporal envelope, this definition no longer provides an accurate description of the signal. To compensate for the finite bandwidth of the signal, we define quadrature amplitudes using a Fourier transform with a window function that matches the temporal envelope of the signal. This window function prevents the quadrature amplitudes from being affected by noise that occurs either before or after the signal. Including the window function in Eqns. 7 gives

$$
\begin{aligned}
X_1 &= \sqrt{\frac{2}{\zeta\mathcal{G}}} \cdot \sqrt{\frac{\Delta t}{C}} \cdot \sum_{k=1}^{N} f(k) \cdot y(k) \cdot \cos(\omega \cdot k \cdot \Delta t) \\
X_2 &= \sqrt{\frac{2}{\zeta\mathcal{G}}} \cdot \sqrt{\frac{\Delta t}{C}} \cdot \sum_{k=1}^{N} f(k) \cdot y(k) \cdot \sin(\omega \cdot k \cdot \Delta t)
\end{aligned} \tag{8}
$$

where $f(k)$ is the temporal envelope of the signal and $C = \sum_{k=1}^{N} |f(k)|^2$ is a factor that compensates for the noise equivalent bandwidth of $f(k)$.

Using the window function $f(k)$ with a Fourier transform is equivalent to using least-squares parameter estimation, where the parameters to be estimated are the amplitudes

of $f(k) \cdot \cos(\omega \cdot k \cdot \Delta t)$ and $f(k) \cdot \sin(\omega \cdot k \cdot \Delta t)$. This method of parameter estimation is appropriate when the noise in the voltage waveform is Gaussian, as is the case for our measurements [9].

We use Eqns. 8 to calculate the quadrature amplitudes X_1 and X_2 that are plotted in Fig. 4a of the main text. Estimates of X_1 and X_2 utilize our knowledge of the converted signal's temporal envelope and center frequency, and also the efficiency and gain of our microwave receiver. We independently determine the efficiency of the microwave receiver using careful calibrations of the microwave receiver (see Supplementary Note 6). Our measured efficiency has an estimated systematic uncertainty of 3%. We account for systematics in the receiver gain by correcting $\mathcal{G}$ so that the calculated variance of X_1 and X_2 (for ω far detuned from the signal's center frequency) has the same variance as vacuum. We estimate the gain has a systematic uncertainty of up to 2%. Assuming the errors in the receiver efficiency and gain are independent, the total systematic error on the added noise is 4%.

SUPPLEMENTARY NOTE 6: CALIBRATION OF MICROWAVE RECEIVER

The microwave receiver employs a Josephson parametric amplifier (JPA) [10]. The JPA amplifies one quadrature of a microwave signal and deamplifies the other, where a quadrature is composed of linear combinations of signals at frequencies above and below the JPA's pump frequency [11]. Before amplification or deamplification, the spectral density of each quadrature emitted from the load resistor is given by, in units of quanta (a quanta corresponding to a photon per second per unit bandwidth),

$$\mathcal{S}_{\mathrm{L}} = \frac{1}{2}\coth\left(\frac{\hbar\omega}{2k_{\mathrm{B}}T_{\mathrm{L}}}\right) \tag{9}$$

where T_{L} is the temperature of the load resistor [12]. At temperatures $T_{\mathrm{L}} \ll \hbar\omega/k_{\mathrm{B}}$, S_{L} approaches 1/2 quantum. At these low temperatures, the total spectral density of both quadratures is thus 1 quantum, consistent with the expectation that each unit of bandwidth has a minimum spectral density of 1/2 quantum [13]. Similarly, at temperatures $T_{\mathrm{L}} \gg \hbar\omega/k_{\mathrm{B}}$, the total power emitted in both quadratures by the resistive load is $2k_{\mathrm{B}}T_{\mathrm{L}}/\hbar\omega$, the classical Johnson noise formula for two units of bandwidth.

Once measured by the microwave receiver, we expect to observe a spectral density given

by [14]

$$\mathcal{S}_{\text{out}} = \mathcal{G}\left(\frac{1-\zeta}{2} + \zeta\mathcal{S}_{\text{L}}\right) \tag{10}$$

where ζ is the efficiency of the receiver and $\mathcal{G}$ is the gain. The efficiency can range from $\zeta = 0$ (no measurement) to $\zeta = 1$ (perfect measurement). Fitting this model to the measured receiver output at $\omega_{\text{e},1} = 2\pi \times 7.08$ GHz yields a gain and efficiency as shown in Supplementary Figure 6.

We use the microwave receiver to measure signals reflected or emitted from the mode converter. These signals are entirely contained in frequencies above the JPA pump frequency. As such, the measured signal is contained neither in the amplified nor deamplified quadrature, but in both. The signal in the deamplified quadrature is not measured and this loss of information reduces the efficiency of our microwave receiver by a factor of two, so for a signal near frequency $\omega_{\text{e},1}$, the efficiency of the receiver is $\zeta/2 = 0.25$. The maximum possible efficiency for this mode of operation is $\zeta/2 = 0.5$.

During the experiment, we operate the microwave receiver around two different frequencies, $\omega_{\text{e},1}$ and $\omega_{\text{e},2}$. The receiver has a frequency dependent performance, so let $\mathcal{G}_1$ ($\mathcal{G}_2$) be the gain and ζ_1 (ζ_2) be the efficiency of the receiver at frequency $\omega_{\text{e},1}$ ($\omega_{\text{e},2}$). These values are summarized in Supplementary Table 2.

To acquire the data in Fig. 3 of the main text, we did not employ the JPA. Without the JPA, the gain and efficiency of the microwave receiver are considerably different, so let $\mathcal{G}_1'$ ($\mathcal{G}_2'$) be the gain and ζ_1' (ζ_2') be the efficiency of the receiver without the JPA at frequency $\omega_{\text{e},1}$ ($\omega_{\text{e},2}$); these values are summarized in Supplementary Table 3. To calculate the photon number conversion efficiency of the mode converter quoted in the main text, we compared the energy in the unconverted signal at $\omega_{\text{e},1}$ and the energy in the converted signal at $\omega_{\text{e},2}$. We calculate a conversion efficiency given by

$$\frac{E_2}{E_1} \times \frac{\mathcal{G}_1'\zeta_1'}{\mathcal{G}_2'\zeta_2'} = \frac{E_2}{E_1} \times 0.60,$$

where E_2 (E_1) is the energy of the converted (unconverted) signal measured at the output of the receiver.

[1] F. Intravaia and A. Lambrecht, "Surface plasmon modes and the Casimir energy," Phys. Rev. Lett. **94**, 110404 (2005).

[2] C. C. Speake and C. Trenkel, "Forces between conducting surfaces due to spatial variations of surface potential," Phys. Rev. Lett. **90**, 160403 (2003).

[3] Robert G. Rogers, *Low Phase Noise Microwave Oscillator Design* (Artech House, Boston, 1991).

[4] Markus Aspelmeyer, Tobias J. Kippenberg, and Florian Marquardt, "Cavity optomechanics," Rev. Mod. Phys. **86**, 1391–1452 (2014).

[5] Jing Zhang, Kunchi Peng, and Samuel L. Braunstein, "Quantum-state transfer from light to macroscopic oscillators," Phys. Rev. A **68**, 013808 (2003).

[6] Jennifer W. Harlow, *Microwave Electromechanics: Measuring and Manipulating the Quantum State of a Macroscopic Mechanical Oscillator*, Ph.D. thesis, Univ. of Colo. at Boulder (2013).

[7] A. Schliesser, R. Riviére, G. Anetsberger, O. Arcizet, and T. J. Kippenberg, "Resolved-sideband cooling of a micromechanical oscillator," Nature Physics **4**, 415–419 (2008).

[8] T. Rocheleau, T. Ndukum, C. Macklin, J. B. Hertzberg, A. A. Clerk, and K. C. Schwab, "Preparation and detection of a mechanical resonator near the ground state of motion," Nature **463**, 72–75 (2010).

[9] Bruce P. Gibbs, *Advanced Kalman Filtering, Least-Squares and Modeling: A Practical Handbook* (John Wiley and Sons, New York, 2011).

[10] M.A. Castellanos-Beltran, K.D. Irwin, G.C. Hilton, L.R. Vale, and K.W. Lehnert, "Amplification and squeezing of quantum noise with a tunable Josephson metamaterial," Nature Physics **4**, 929–931 (2008).

[11] B. Yurke, L. R. Corruccini, P. G. Kaminsky, L. W. Rupp, A. D. Smith, A. H. Silver, R. W. Simon, and E. A. Whittaker, "Observation of parametric amplification and deamplification in a Josephson parametric amplifier," Phys. Rev. A **39**, 2519–2533 (1989).

[12] Manuel Castellanos-Beltran, *Development of a Josephson Parametric Amplifier for the Preparation and Detection of Nonclassical states of Microwave Fields*, Ph.D. thesis, Univ. of Colo. at Boulder (2010).

[13] A. A. Clerk, M. H. Devoret, S. M. Girvin, Florian Marquardt, and R. J. Schoelkopf, "Intro-

duction to quantum noise, measurement, and amplification," Rev. Mod. Phys. **82**, 1155–1208 (2010).

[14] T. A. Palomaki, J. D. Teufel, R. W. Simmonds, and K. W. Lehnert, "Entangling mechanical motion with microwave fields," Science **342**, 710–713 (2013).
